# Three-dimensional morphologies, substructures, and crystallography of pearlite in carbon steel

**DOI:** 10.1080/14686996.2025.2523242

**Published:** 2025-06-26

**Authors:** Akinobu Shibata, Akiko Nakamura, Taku Moronaga, Kazuho Okada, Toru Hara, Rintaro Ueji

**Affiliations:** a Research Center for Structural Materials, National Institute for Materials Science (NIMS), Tsukuba, Japan; b Research Network and Facility Services Division, National Institute for Materials Science (NIMS), Tsukuba, Japan

**Keywords:** Pearlitic steel, microstructure formation mechanism, transmission electron microscopy, serial-sectioning, crystallography

## Abstract

The present study examined the morphology, substructure, crystallography, and element distribution of as-transformed (air-cooled) pearlite in medium-carbon steel using focused ion beam-scanning electron microscopy serial sectioning and advanced transmission electron microscopy analysis. The three-dimensional analysis revealed that the cementite did not exhibit a fully continuous lamellar structure, and the long axis direction of non-continuous region was nearly identical within each cementite lamella and among the adjacent cementite lamellae. The measured macroscopic interfacial plane orientation ranged from (0 1 0)_θ_ to ($\bar{1}$ 2 0)_θ_ and ($\bar{2}$ $\bar{1}$ 5)_α_ to ($\bar{1}$ $\bar{1}$ 1)_α_. The growth directions of cementite lamellae tended to align with the invariant line between cementite and ferrite, as well as the parallel direction in the Pitsch-Petch relationship. Even within a single colony, the orientations of both the ferrite and cementite regions changed discontinuously, forming low-angle boundaries, some of which exhibited a staircase-like shape. The orientation relationship between ferrite and cementite changed slightly at the low-angle boundary within a colony. This indicates that when the accumulated misfit strain exceeds a certain value, the parallel direction relationship changes to accommodate the accumulated strain while maintaining a nearly identical orientation relationship. The concentration inside cementite lamella was not completely homogeneous; manganese and chromium were enriched, while carbon was depleted, at the lamellar interface. We surmised that the inhomogeneous element distribution in cementite lamella could be attributed to the incomplete partitioning behavior of alloying elements at transformation, as well as their segregation at the lamellar interface, aiming to reduce interfacial energy.

## IMPACT STATEMENT

The present paper provides a comprehensive understanding of the formation mechanism of pearlite structures in steel by employing state-of-the-art techniques, including FIB-SEM serial sectioning and advanced TEM analysis, notably ACOM-TEM.

## GRAPHICAL ABSTRACT

**Figure:**
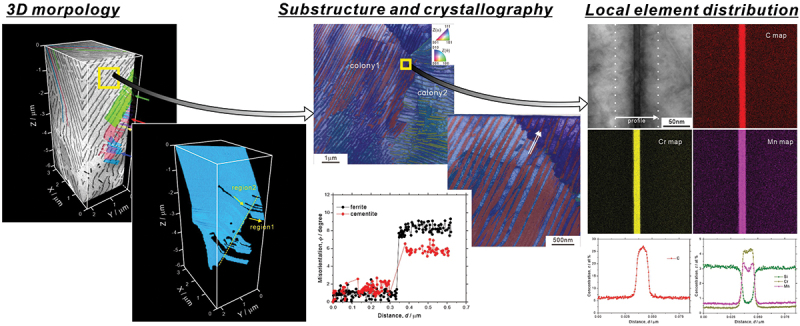

## 1. Introduction

Pearlite, formed in medium- and high-carbon steels, is one of the most important microstructures in engineering steels due to its ability to provide high strength. For example, cold-drawn pearlite steel wires have an extremely high strength up to 5–6 GPa [1–3]. Additionally, the as-transformed pearlite itself exhibits high resistance to wear, fatigue fracture, and hydrogen embrittlement [4–7]. However, low impact-toughness is one of the critical issues for the application of pearlite steels [8–11]. The characteristic microstructural feature of pearlite is a lamellar structure consisting of alternating ferrite and cementite lamellae. Ferrite exhibits body-centered cubic structure and hardly dissolves carbon, while cementite, Fe_3_C, contains a large amount of carbon and has an orthorhombic unit cell corresponding to the space group of *P*_*nma*_ (given the lattice parameters along the [1 0 0], [0 1 0], and [0 0 1] directions in cementite as *a*, *b*, and *c*, respectively, the convention *c* < *a* < *b* is adapted in the present study). In addition to the lamellar structure, pearlite consists of several structural units, namely nodule and colony [12]. Through a pearlite transformation, an austenite grain is divided into several nodules, which are units with nearly identical orientation of ferrite. Inside a nodule, there are several colonies, and the colony is defined as the region where the interface normal of cementite lamella is identical.

Although there is a certain understanding of the pearlite microstructure, a characteristic heterogeneity in morphology and orientation distribution has also been observed (examples are described below) [13–22], indicating that the microstructural study of pearlite is still in progress. Adachi et al. [18,21] studied three-dimensional (3D) morphology of cementite in a eutectoid Fe-C alloy by focused ion beam-scanning electron microscopy (FIB-SEM) serial sectioning. They showed that the cementite did not exhibit a complete lamellar shape. Moreover, some of the cementite lamellae were twisted in three-dimensions. They proposed that the non-continuous regions in naturally grown cementite lamella led to the shape instability and induced the initiation and development of cementite spheroidization. Several studies also reported the existence of ferrite intrusions in a cementite lamella [13–15], though their formation mechanism remains uncertain.

To date, three main orientation relationships have been recognized for ferrite and cementite in pearlite (in the following, the subscripts θ and α indicate cementite and ferrite, respectively); the Pitsch–Petch relationship [23,24]

$\left({\left[1\;0\;0\right]}_{\theta }∼\;{\left[1\;3\;1\right]}_{\alpha },{\left(0\;1\;0\right)}_{\theta }//\;(\overset{ˉ}{2}\;\overset{ˉ}{1}\;5{)}_{\alpha },\;{\left[0\;0\;1\right]}_{\theta }\text{\textbackslash{}break}∼\;[3\;\;\overset{ˉ}{1}\;1{]}_{\alpha }\right)$, Bagaryatskii relationship [25], ([1 0 0]_θ_ // [1 1 1]_α_, (0 1 0)_θ_ // $(\overset{ˉ}{1}\;\overset{ˉ}{1}\;2{)}_{\alpha }$, [0 0 1]_θ_ // $[1\;\overset{ˉ}{1}\;0{]}_{\alpha }$) and Isaichev relationship[26] ${\left[1\;0\;0\right]}_{\theta }//\;{\left[1\;1\;1\right]}_{\alpha },$${\left(0\;1\;1\right)}_{\theta }//\;{\left(\bar{1}\;\bar{1}\;2\right)}_{\alpha },{\left[0\;0\;1\right]}_{\theta }//\;{\left[1\;\bar{1}\;0\right]}_{\alpha })$. It has been reported that the pearlite nucleated on clean austenite grain boundary, typically in eutectoid composition, exhibits the Pitsch–Petch relationship [27,28]. On the other hand, the Bagaryatskii and Isaichev relationships have been found for pearlite nucleated from hyper-eutectoid cementite and pro-eutectoid ferrite, respectively [27,28]. These three relationships can sometimes be simultaneously established in a given pearlite structure [22]. Zhou and Shiflet [28] observed the lattice images of pearlite in an Fe-12Mn-0.81C alloy and reported that the atomistic habit planes of cementite, which are the planes of interface between ferrite and cementite, basically corresponded to the planes holding parallel relationship: ${\left(0\;1\;0\right)}_{\theta }//(\overset{ˉ}{2}\;\overset{ˉ}{1}\;5{)}_{\alpha }$ in the Pitsch–Petch relationship, ${\left(0\;1\;0\right)}_{\theta }//\;(\;\overset{ˉ}{1}\;\overset{ˉ}{1}\;2{)}_{\alpha }$ in the Bagaryatskii relationship, and ${\left(0\;1\;1\right)}_{\theta }//\;(\;\overset{ˉ}{1}\;\overset{ˉ}{1}\;2{)}_{\alpha }$ in the Isaichev relationship. On the other hand, Zaefferer et al. [19] reported via 3D analysis using FIB-SEM serial sectioning that the interfacial plane of cementite lamella does not exhibit a unique orientation. However, they focused only on the orientation of the ferrite region and did not identify the orientation of the cementite region. According to the traditional definition of structural unit of pearlite described above, orientation of ferrite inside a nodule is uniform. However, the recent studies revealed that there is a slight misorientation across the adjacent colonies [16–19]. Walentek et al. [16] reported that the ferrite matrix contained a network of low-angle boundaries even in one colony. In contrast, Nakada et al. [20] insisted that the misorientations of ferrite as well as cementite increased continuously along a cementite lamella, attributing this crystallographic orientation rotation to elastic strain originating from the misfit at the ferrite/cementite interface. Recently, phase-field simulations have been utilized to understand the transformation kinetics, local element distribution at growth fronts in the three-phase region (austenite/ferrite/cementite), and development of lamellar structures [29–33].

While extensive experimental and simulation research has been conducted on pearlite structures, further exploration of the substructure (i.e. intrinsic defects) and crystallography is essential for a thorough understanding of the formation mechanism of pearlite structures. Fundamental research on microstructure in pearlite can offer insights into developing novel microstructural control strategies aimed at enhancing advanced high-strength steels. Recently, we developed an analysis method that connects the 3D morphology of microstructures, with crystallography using FIB-SEM serial sectioning and used this method to study the formation mechanism of microstructure as well as the relationship between microstructure and fracture behavior in martensitic steels [34–37]. Applying this approach to the pearlite microstructure in steels is expected to yield new insights. Although several recent studies have utilized electron backscattering diffraction (EBSD) with SEM to analyze the orientation of cementite, specifically in larger sizes [16,20], the conventional techniques have primarily relied on selected area diffraction analysis in transmission electron microscopy (TEM). In addition to the SEM-EBSD technique, orientation mapping with precession electron diffraction in TEM (known as automated crystallographic orientation mapping in TEM, ACOM-TEM) can provide high spatial resolution orientation and phase maps of ferrite and cementite in a pearlite structure. The present study revisits the morphologies, substructures, and crystallography of pearlite by utilizing FIB-SEM serial sectioning and advanced TEM techniques (such as ACOM-TEM).

## 2. Experimental procedures

This study used a medium-carbon steel containing manganese, silicon, and chromium. The detailed chemical composition was C: 0.62%, Si: 2.02%, Mn: 0.23%, P: 0.001%, S: <0.001%, Cr: 1.01%, and Fe: balance (mass%). A round bar with dimensions of 40 mm diameter and 100 mm length was austenitized at 850°C for 30 min, followed by air cooling. The heat-treated specimen consists of pearlite and a small amount of pro-eutectoid ferrite [38]. The following analyses were conducted at the center of the specimen.

Microstructures were observed using SEM with an acceleration voltage of 2 kV (Hitachi High-Tech, SMF-1000) and TEM/scanning transmission electron microscopy (STEM) operated at 200 kV and 300 kV (JEOL, JEM-2800 and JEM-ARM300F). Thin foils for TEM/STEM analysis were fabricated using an FIB (ThermoFisher Scientific, Scios 2). Local crystal orientation mappings for the fabricated thin foils were performed using ACOM-TEM (NanoMEGAS, ASTAR). The measurement areas were 3.2 μm × 3.2 μm and 8 μm × 8 μm, with step sizes of 2 nm and 5 nm, respectively. Local element distribution was analyzed using STEM equipped with energy-dispersive X-ray spectroscopy (EDX). The EDX measurements were performed under the edge-on condition of cementite lamella, which was determined through careful tilting experiments in STEM.

A pillar with dimensions of approximately 25 μm × 25 μm × 25 μm was lifted out from the specimen using an FIB, and the serial sectioning was performed using an orthogonal FIB-SEM system (Hitachi High-Tech, SMF-1000) [39]. The pillars were milled at intervals of 20 nm with an acceleration voltage of 30 kV and a beam current of ~6 nA. The microstructure of each milled surface was observed using SEM with an acceleration voltage of 2 kV. The ORS Dragonfly software was used to reconstruct the 3D images from the series of SEM images. A detailed procedure for preparing the specimen for FIB-SEM serial sectioning and TEM/STEM observation is described in Appendix A.

## 3. Results

### 3.1. Orientation relationship between ferrite and cementite

**Figure 1.**
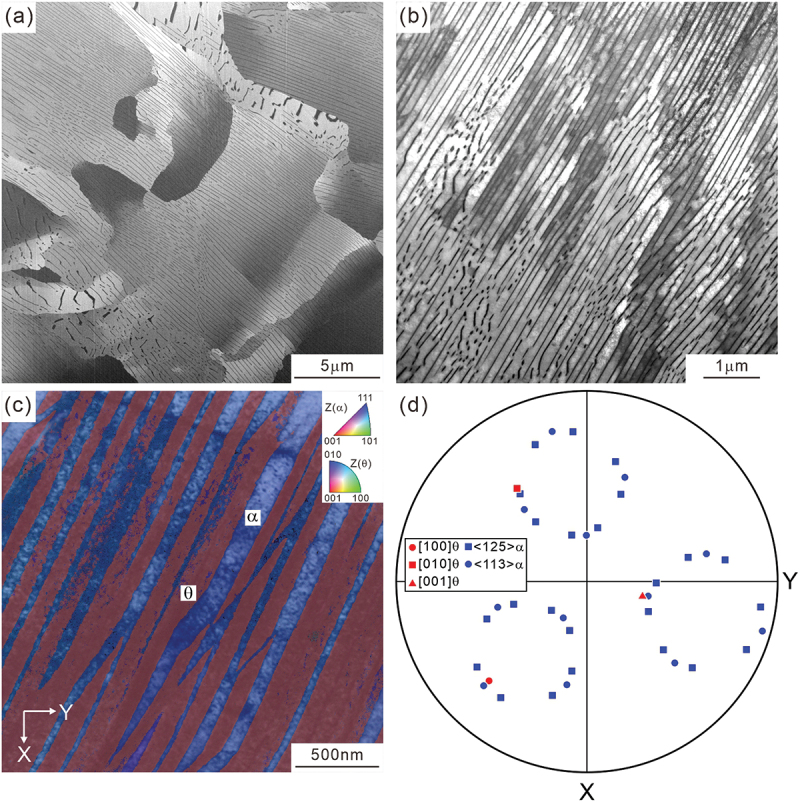
(a) SEM image, (b) STEM image, (c) ACOM-TEM orientation map superimposed on the STEM image, and (d) pole figure of ferrite (<1 2 5>_α_, <1 1 3>_α_, blues) and cementite ([1 0 0]_θ_, [0 1 0]_θ_, [0 0 1]_θ_, red) in (c).

As shown in the SEM and STEM images of Figure 1(a,b), the heat-treated specimen exhibits a typical pearlite with a fine lamellar structure of ferrite and cementite. Figure 1(c) illustrates an ACOM-TEM orientation map superimposed on the STEM image, demonstrating that ACOM-TEM analysis can identify the local crystallographic orientation of the cementite with a high spatial resolution. The color indicates a crystallographic orientation parallel to the normal direction of the observed section according to the color map inserted at the right upper corner. Because the ACOM-TEM measurement was performed under a no-tilt condition and the cementite lamellae are inclined to the incident electron beam direction, the cementite lamellae appear to be somewhat thicker in the image. The pole figure obtained by ACOM-TEM (Figure 1(d)) indicates that the cementite holds the Pitsch–Petch relationship with respect to the surrounding ferrite; ([1 0 0]_θ_ ~ [1 3 1]_α_ (red circle and blue circle), $[0\;1\;0{]}_{\theta }//\;[\overset{ˉ}{2}\;\overset{ˉ}{1}\;5{]}_{\alpha }$ (red rectangle and blue rectangle), and ${\left[0\;0\;1\right]}_{\theta }∼\;[3\;\;\overset{ˉ}{1}\;1{]}_{\alpha }$ (red triangle and blue circle). All the observed orientation relationship in the analyzed areas is the Pitsch–Petch relationship. The following crystallographic analysis is based on this variant of the Pitsch–Petch relationship.

### 3.2. Morphology of cementite determined by 3D analysis

**Figure 2.**
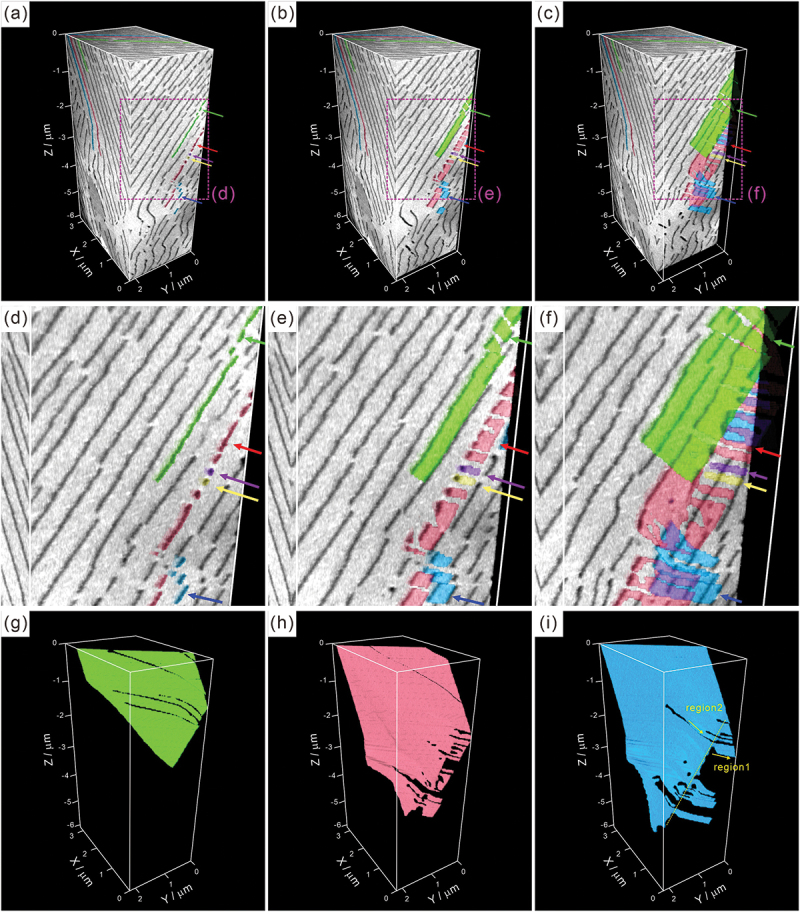
3D morphology of pearlite on each section: (a,d) *X* = 0 μm, (b,e) *X* = 0.085 μm, and (c,f) *X* = 0.355 μm, and (g–i) each extracted cementite regions ((d–f) are enlarged views of the areas indicated by dashed rectangles in (a–c)).

The 3D analysis using FIB-SEM serial sectioning is illustrated in Figure 2. The cementite regions in the analyzed volume were segmented by machine learning, with the U-net function using the ORS Dragonfly software, and each color represents the individual connected cementite region. In Figure 2(a,d), the colored arrows (green, red, purple, yellow, and blue) indicate the fragmented and isolated cementite regions on the *YZ* section of *X* = 0 μm. Particularly, the cementite regions indicated by purple and yellow arrows exhibit a round shape. One may consider that spheroidization of cementite occurred partially. However, as shown in the *YZ* sections of *X* = 0.085 μm (Figure 2(b,e)) and *X* = 0.355 μm (Figure 2(c,f)), these cementite regions are not spheroidized; instead, they exhibit a rod-like shape. Moreover, the cementite regions which are observed to be fragmented on a given section (for example, the regions indicated by green, red, and blue arrows in Figure 2(a,d)) are actually not isolated but are part of a large lamella. Figure 2(g–i) depict the morphology of each extracted cementite region. The cementite lamella is not fully continuous, and it should be noted that the long axis direction of the non-continuous region where ferrite protrudes into cementite lamella is nearly identical in each cementite lamella as well as in the neighboring cementite lamellae. Moreover, as illustrated in Figure 2(i), for an example, those directions differ between regions 1 and 2. This implies that the long axis direction of the non-continuous region could be parallel to the growth direction of the cementite lamella, and that the direction of a given cementite lamella may intermittently change. The detailed morphology of cementite in Figure 2(i) is presented in the Supplementary Materials section (**Movie S1**).

**Figure 3.**
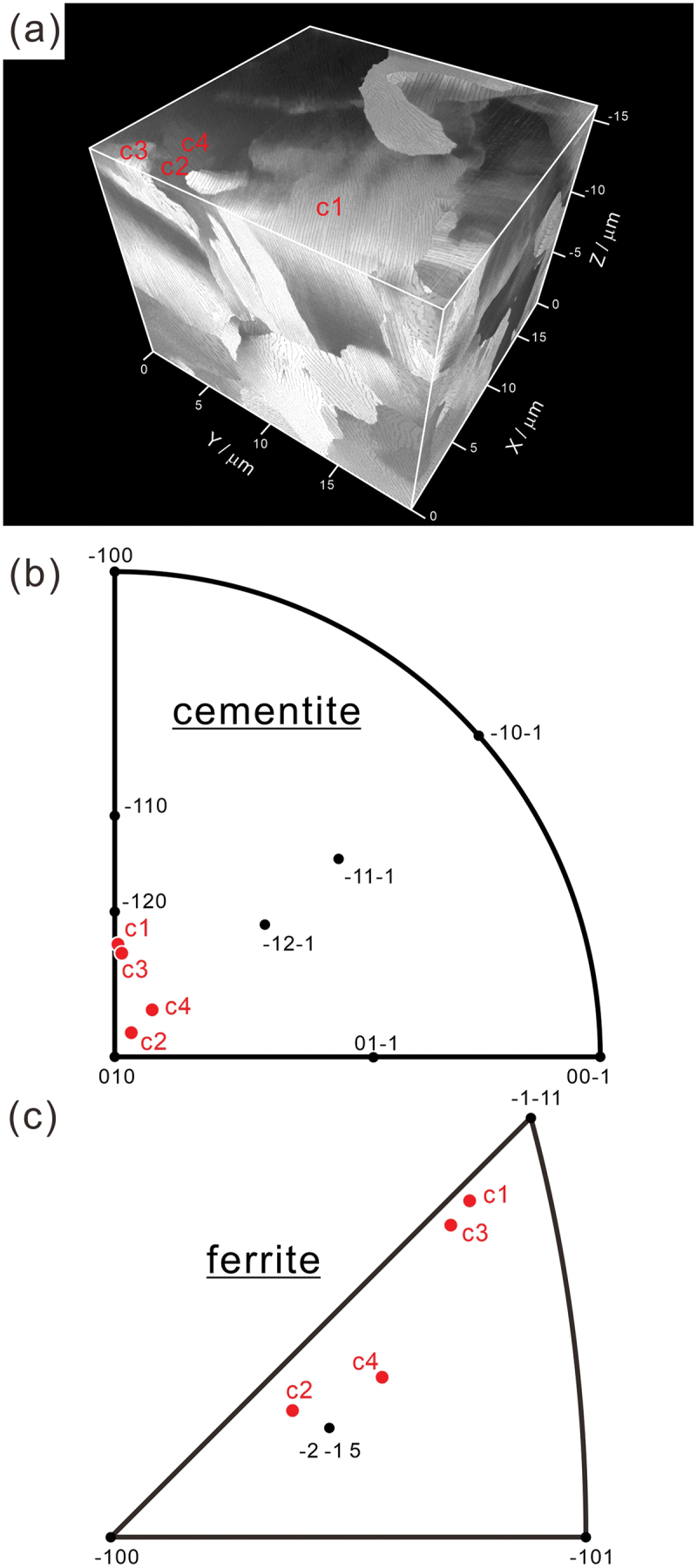
(a) 3D image of pearlite and (b,c) interfacial plane orientation with respect to cementite and ferrite coordinate systems.

**Figure A1.**
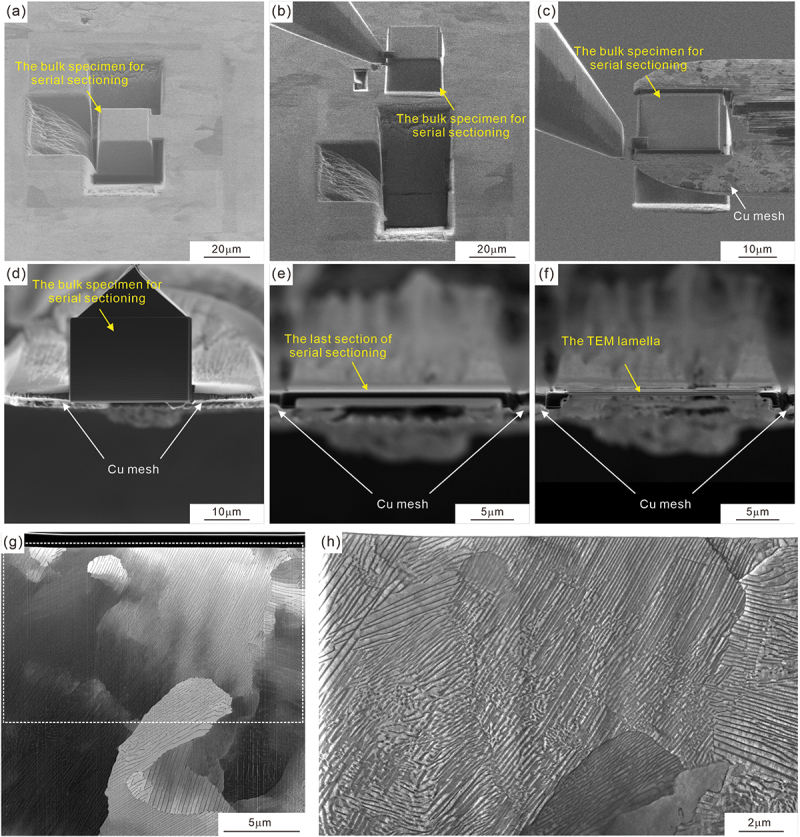
The detailed procedure for preparing the specimen for FIB-SEM serial sectioning and TEM/STEM observation: (a–c) SEM images showing (a) the specimen after FIB machining, (b) after the specimen was lifted out, and (c) after it was mounted onto the copper mesh. (d–f) Scanning ion microscopy (SIM) images showing (d) the specimen before FIB-SEM serial sectioning, (e) the specimen after FIB-SEM serial sectioning, and (f) the specimen for TEM/STEM observation after FIB milling. (g,h) Identical area observation results of (g) the SEM image of the final section of FIB-SEM serial sectioning and (h) the STEM image (the white rectangle in (g) corresponds to the observed area of (h)).

The orientation of the interfacial plane of cementite lamella was determined through two-surface trace analysis using a 3D image, and the results are presented in Figure 3; (a) 3D image of pearlite and (b, c) interfacial plane orientations with respect to cementite and ferrite coordinate systems, respectively. Because we fabricated the TEM/STEM thin-foil from the region near the final section of FIB-SEM serial sectioning and performed ACOM-TEM analysis, as shown in Figure A1(e,f) in Appendix A, the crystallographic orientations of ferrite and cementite determined by ACOM-TEM were utilized for two-surface trace analysis. The sectioning direction in the analyzed volume is -*Z* direction, meaning that the final section where ACOM-TEM analysis was performed corresponds to the top surface in Figure 3(a). Because each cementite lamella does not exhibit a fully continuous morphology (Figure 2), a two-surface trace analysis was conducted on the apparent lamellar-shaped regions, using the average inclination over a length of approximately 1–4 micrometers in the depth direction on the ACOM-TEM observation plane. As shown in Figure 3(b,c), the interfacial planes of cementite lamella in colony 2 (c2) and colony 4 (c4) are close to (0 1 0)_θ_ and $(\overset{ˉ}{2}\;\overset{ˉ}{1}\;5{)}_{\alpha }$ These planes correspond to the parallel planes of the Pitsch-Petch relationship. On the other hand, the interfacial planes of cementite lamella in colony 1 (c1) and colony 3 (c3) are rather parallel to $\left(\bar{1}\;2\;0\right)$_θ_ and $(\overset{ˉ}{1}\;\overset{ˉ}{1}\;1{)}_{\alpha }$. Because each cementite lamella in colonies 1 ~ 4 maintains the Pitsch-Petch relationship with the surrounding ferrite, we can infer that the crystallographic orientation of the macroscopic interfacial plane of cementite is not unique, unlike the case of martensite and bainite. The interfacial plane orientation varies within a range from (0 1 0)_θ_ to $\left(\bar{1}\;2\;0\right)$_θ_ and $(\overset{ˉ}{2}\;\overset{ˉ}{1}\;5{)}_{\alpha }$ to $(\overset{ˉ}{1}\;\overset{ˉ}{1}\;1{)}_{\alpha }$. Zaefferer et al. [19] also reported that there was no clear relationship between the crystal orientation and the macroscopic interfacial plane.

**Figure 4.**
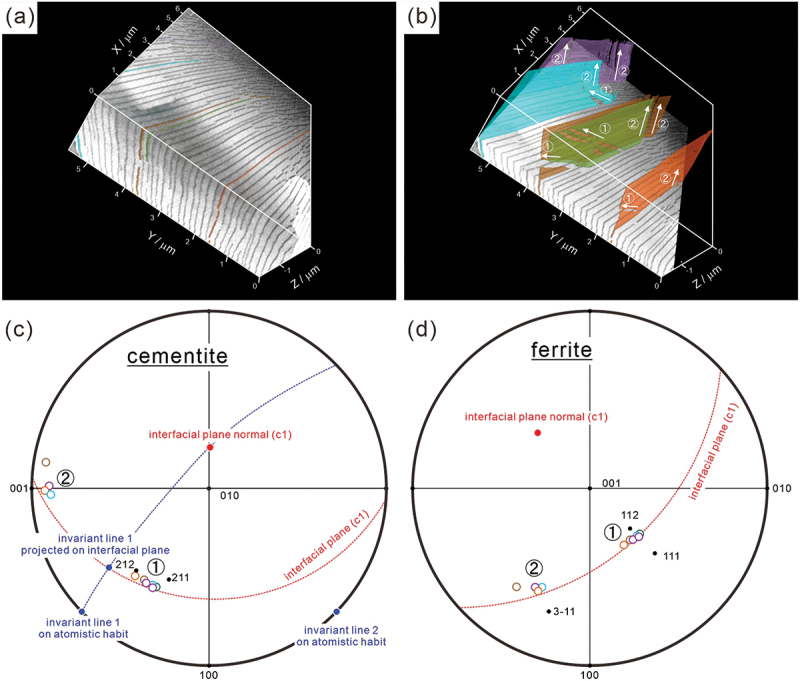
(a,b) 3D images of pearlite and (c,d) standard stereographic projections showing the long axis directions of the non-continuous region of cementite lamella with respect to cementite and ferrite coordinate systems. The invariant lines between cementite and ferrite lamellae, as well as the macroscopic interfacial plane normal, are also plotted in (c,d).

Figure 4(a,b) present high-magnification 3D images of colony 1 in Figures 3(a), with 4(b) specifically illustrating several independent cementite lamellae distinguished by different colors. We should note that all the cementite lamellae share the similar long axis directions of the non-continuous region. This suggests that the growth directions are almost the same in these cementite lamellae (along 1 and 2). The growth directions (the long axis directions of the non-continuous region) were determined using the orientation of cementite and ferrite measured by ACOM-TEM and plotted on the standard stereographic projections of Figure 4(c,d) (the colors of the plots correspond to those of cementite lamellae in (a,b). Growth direction of ‘1’ ranges from [2 1 1]_θ_ to [2 1 2]_θ_ and around [1 1 2]_α_, while growth direction of ‘2’ is primarily around [0 0 1]_θ_ and $[3\;\overset{ˉ}{1}\;1{]}_{\alpha }$. The relationship between the growth direction, invariant line, and interfacial plane depicted in Figure 4(c,d) will be explained in the following discussion section.

### 3.3. Substructure and local element distribution characterized by TEM/STEM

**Figure 5.**
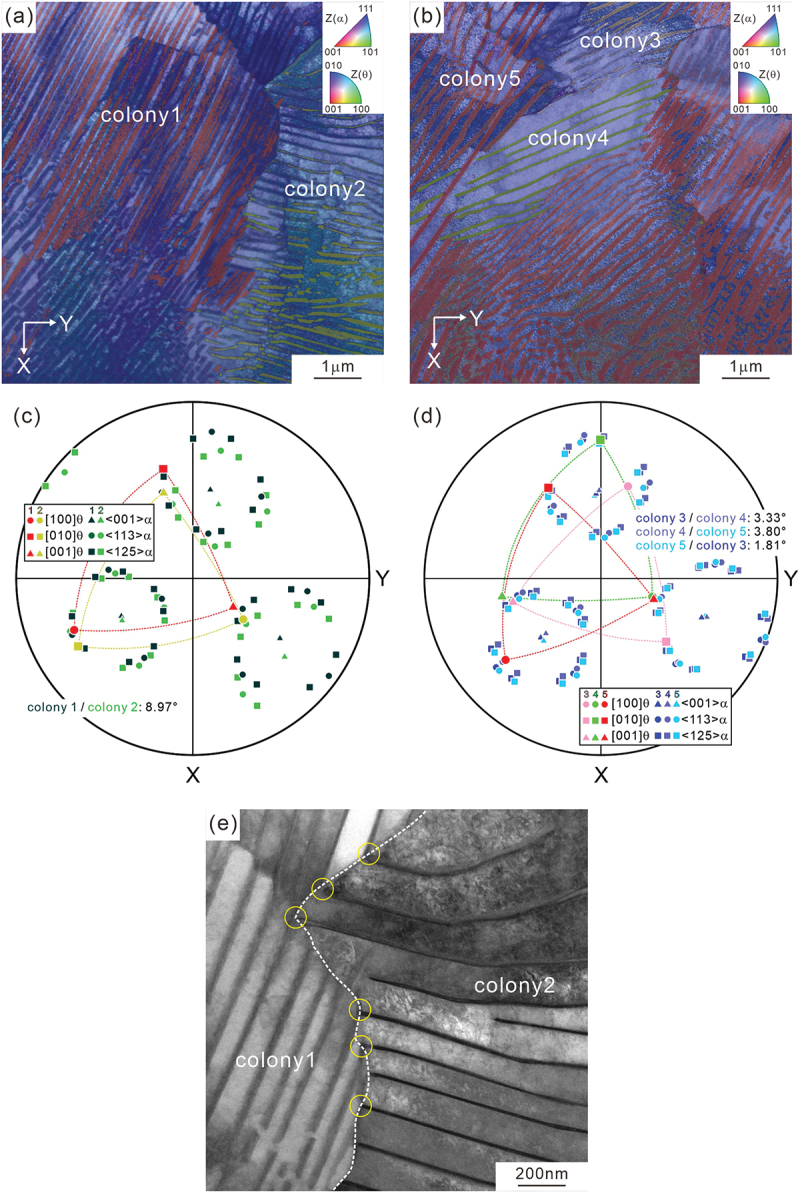
(a,b) ACOM-TEM orientation maps superimposed on the STEM images, (c,d) pole figures of ferrite (<0 0 1>_α_, <1 2 5>_α_, <1 1 3>_α_) and cementite ([1 0 0]_θ_, [0 1 0]_θ_, [0 0 1]_θ_) in (a,b), respectively, and (e) STEM image.

Figure 5(a,b) show ACOM-TEM orientation maps and (c,d) corresponding pole figures. There are five colonies in the observed areas, namely colony 1, 2, 3, 4, and 5, and the cementites in all the colony have the Pitsch–Petch relationship with respect to the surrounding ferrite. The pole figure of Figure 5(c) indicates that the neighboring colonies 1 and 2 in Figure 5(a) exhibit the orientation relationship of [1 0 0]_θ1_ ~ [0 1 0]_θ2_, [0 1 0]_θ1_ ~ [0 0 1]_θ2_, [0 0 1]_θ1_ ~ [1 0 0]_θ2_. For the neighboring three colonies (colony 3, 4, and 5) in Figure 5(b), the relationships of [0 0 1]_θ3_ ~ [0 0 1]_θ4_, and [1 0 0]_θ4_ ~ [0 0 1]_θ5_ can be confirmed, as depicted in Figure 5(d). Furthermore, cementites in colonies 3, 4 and 5 also appear to exhibit a kind of mirror symmetry relationship with each another. Consequently, we can propose that the cementite lamellae in the adjacent colonies have a certain crystallographic orientation relationship, though their interface normals are different from each other. In addition, the orientation of ferrite between colonies has slight misorientation ranging from 2° to 9°. As depicted in the STEM image around the boundary between colonies 1 and 2 in Figure 5(e), some of the cementite lamellae belonging to the different colonies are connected at the colony boundary (yellow circles). These observation results suggest that cementite lamellae in the neighboring colonies did not form independently but rather exhibit a crystallographic orientation relationship, connecting each other at the colony boundary.

**Figure 6.**
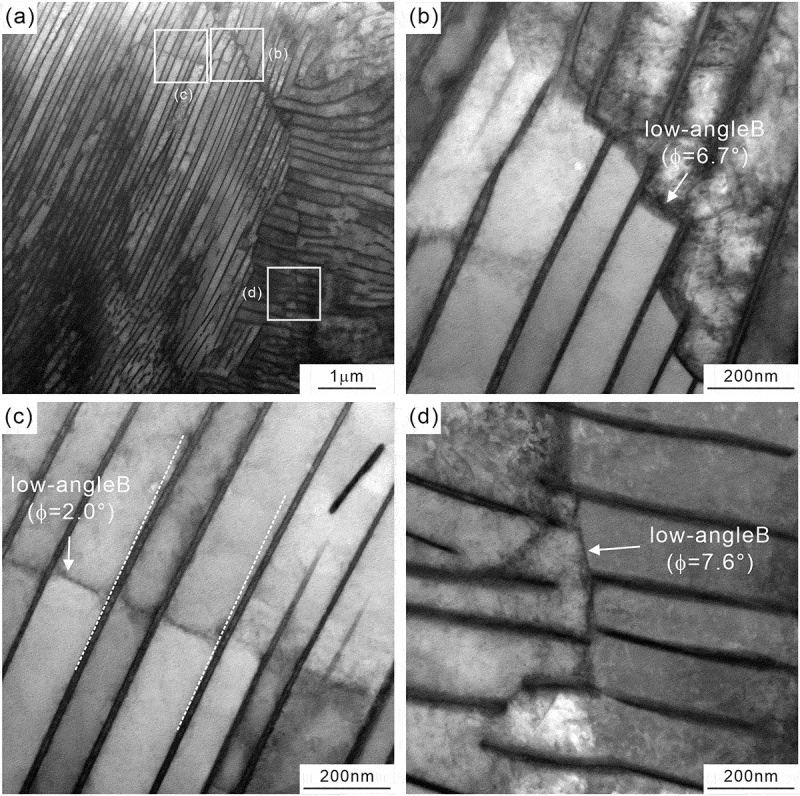
(a) Low-magnification STEM image and (b–d) high magnification STEM images whose observation areas are indicated in (a).

**Figure 7.**
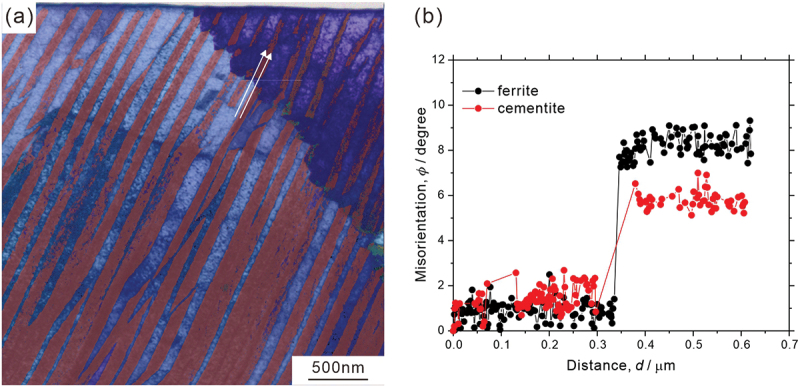
(a) ACOM-TEM orientation map superimposed on the STEM image and (b) misorientation profiles along the white arrows indicated in (a).

**Figure 8.**
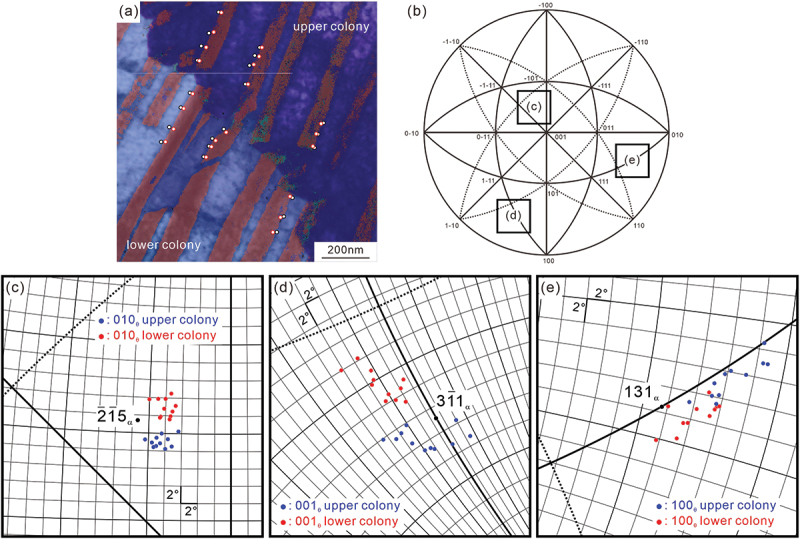
(a) ACOM-TEM orientation map superimposed on the STEM image and (b – e) standard stereographic projection of ferrite on which the poles of [0 1 0]_θ_ ((c), around $[\overset{ˉ}{2}\;\overset{ˉ}{1}\;5{]}_{\alpha }$), [0 0 1]_θ_ ((d), around $[3\;\overset{ˉ}{1}\;1{]}_{\alpha }$), and [1 0 0]_θ_ ((e), around [1 3 1]_α_) are plotted.

Figure 6 shows (a) low-magnification and (b–d) high-magnification STEM images, and the observed areas in (b–d) are indicated by the white rectangles in (a). We can observe several boundaries inside a given colony. The misorientation angles of the boundaries measured by ACOM-TEM in the same area are 6.7°, 2.0°, and 7.6° in Figure 6(b-d), respectively, indicating that the orientation inside the colony is not uniform, but changes discontinuously through the formation of low-angle boundaries. In Figure 6(b,d), the cementite lamellae are basically discontinuous at the low-angle boundaries. On the other hand, the cementite lamellae in Figure 6(c) are continuous but slightly change the interface normal at the low-angle boundaries as indicated by the white broken lines. These low-angle boundaries appear as boundaries characterized by a high density of dislocations, similar to the dislocation boundaries in deformed metals [40]. Additionally, we should note that the low-angle boundary in Figure 6(b) exhibits a staircase-like shape. The results of the local orientation analysis are depicted in Figures 7 and 8. Figure 7 shows (a) ACOM-TEM orientation map and (b) misorientation profiles of ferrite and cementite along the white lines in (a). The discontinuous orientation changes at the boundary can be confirmed for both ferrite and cementite (~7° in the ferrite and ~6° in the cementite). The localized arrangement of dislocations (dislocation boundaries) and the coordinated change in the local orientation of ferrite and cementite, as shown in Figures 6 and 7, indicates that local plastic deformation occurs during the pearlite transformation. Figure 8 is the analysis results on the local orientation relationship between ferrite and cementite; (a) ACOM-TEM orientation map and (b–e) standard stereographic projection of ferrite on which the poles of [0 1 0]_θ_ ((c), around $[\overset{ˉ}{2}\;\overset{ˉ}{1}\;5{]}_{\alpha })\;,\;{\left[0\;0\;1\right]}_{\theta }(\left(d\right),\mathrm{around}\;[3\;\overset{ˉ}{1}\;1{]}_{\alpha })$, and [1 0 0]_θ_ ((e), around [1 3 1]_α_) are plotted. The misorientation of the ferrite regions in the upper and lower colonies is 6.7° (Figure 6(b)). The orientations of neighboring ferrite and cementite, as indicated by circle markers in Figure 8(a), were used to transform the orientation of cementite into the coordinate system of ferrite. A detailed method to plot the cementite orientation on the stereographic projection of ferrite coordinate system is described in Appendix B. For the Pitsch–Petch relationship, it is impossible to simultaneously achieve both ${\left[0\;0\;1\right]}_{\theta }//\;[3\;\overset{ˉ}{1}\;1{]}_{\alpha }$ and ${\left[1\;0\;0\right]}_{\theta }\;//\;\;[1\;3\;\;1{]}_{\alpha }$, as $[3\;\overset{ˉ}{1}\;1{]}_{\alpha }$ and $[1\;3\;\;1{]}_{\alpha }$ are not orthogonal. As shown in Figure 8(c–e), the upper colony (blue plots) exhibits ${\left[0\;0\;1\right]}_{\theta }//\;[3\;\overset{ˉ}{1}\;1{]}_{\alpha }$, while the lower colony (red plots) satisfies rather [1 0 0]_θ_//[1 3 1]_α_. The misorientation between ${\left[0\;1\;0\right]}_{\theta }$ and $[\overset{ˉ}{2}\;\overset{ˉ}{1}\;5{]}_{\alpha }$ are almost the same between the upper and lower colonies. These results indicate that orientation relationship between ferrite and cementite is slightly changed at the low-angle boundary inside a colony.

**Figure 9.**
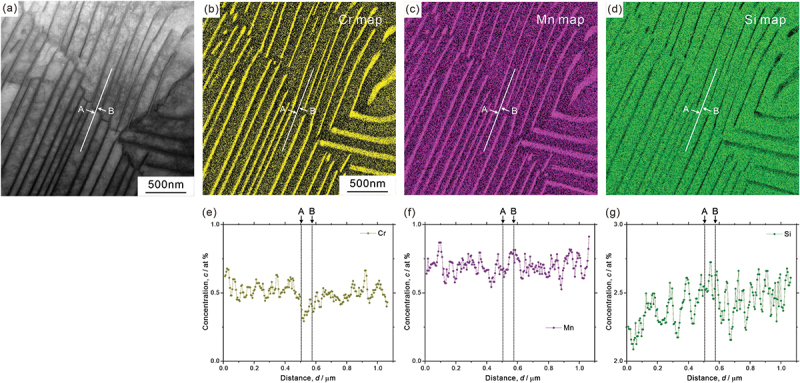
(a) STEM image, (b–d) element distribution maps, and (e–g) concentration profiles of ferrite region ((b,e) chromium, (c, f) manganese, and (d,g) silicon).

**Figure 10.**
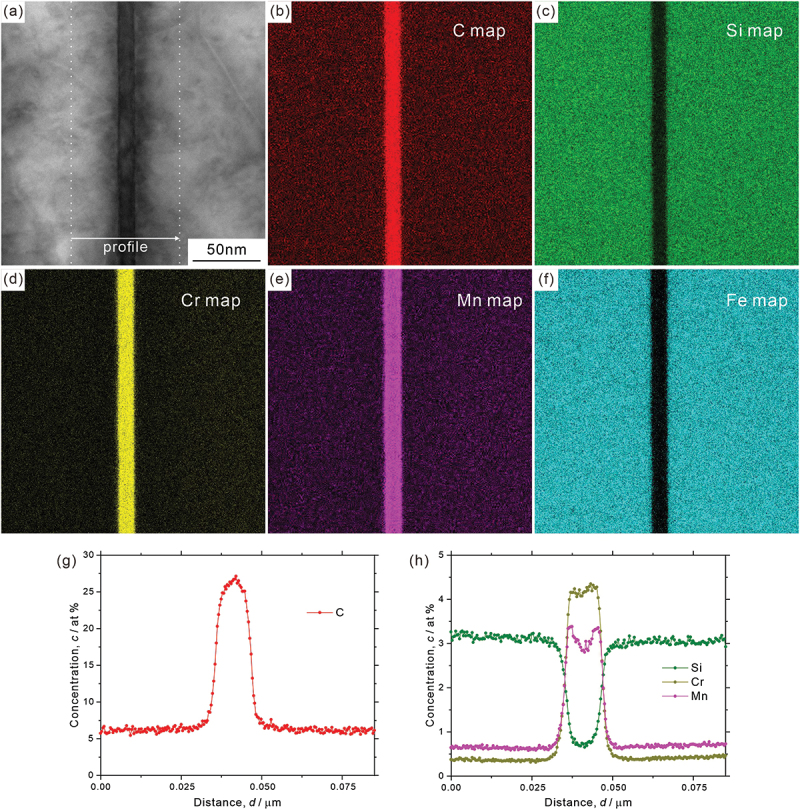
(a) STEM image, (b–f) element distribution maps ((b) carbon, (c) silicon, (d) chromium, (e) manganese, and (f) iron), and (g,h) concentration profiles across the cementite lamella.

**Figure 11.**
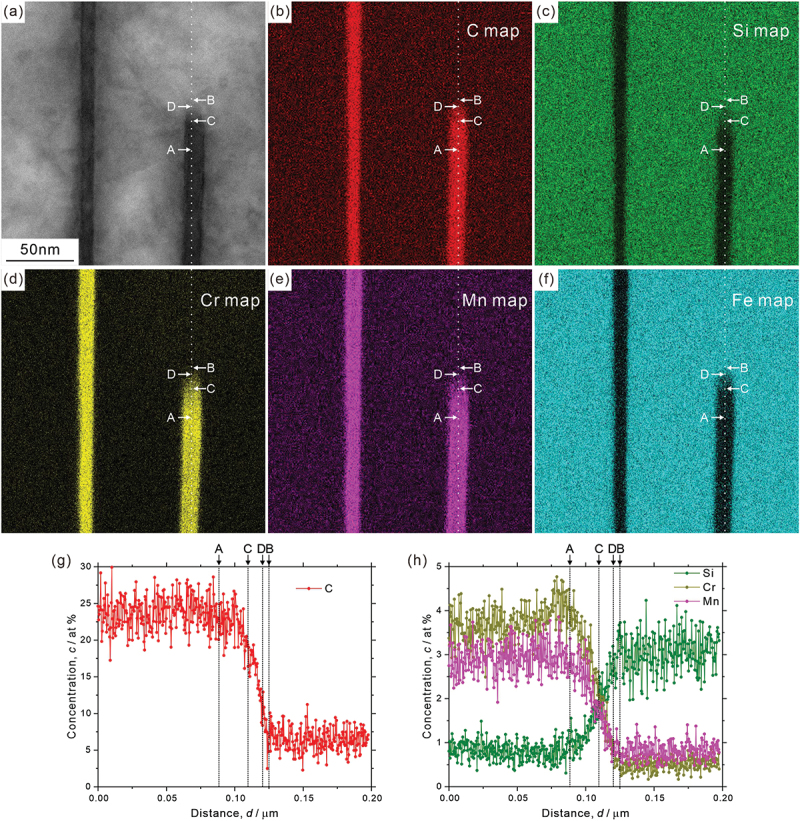
(a) STEM image, (b–f) element distribution maps ((b) carbon, (c) silicon, (d) chromium, (e) manganese, and (f) iron), and (g, h) concentration profiles across the side surface of cementite lamella (corresponding to the growth front at transformation).

Analysis results on element distribution at macroscopic scale are depicted in Figure 9; (a) STEM image, (b–d) element distribution maps, and (e–g) concentration profiles of ferrite region ((b,e) chromium, (c, f) manganese, and (d,g) silicon). The concentration profiles of Figure 9(e–g) were obtained along the white lines indicated in Figure 9(a–d), and ‘A’ and ‘B’ are the positions across the low-angle boundary. Though not so obvious in manganese distribution, we can find that chromium is depleted, while silicon is enriched around the low-angle boundary as illustrated in Figure 9(b–g). Figure 10 presents (a) STEM image, (b–f) element distribution maps ((b) carbon, (c) silicon, (d) chromium, (e) manganese, and (f) iron), and (g,h) concentration profiles across a single cementite lamella. It is clear that carbon, chromium, and manganese are enriched, while silicon and iron are depleted at the cementite. However, the concentration inside the cementite lamella is not completely homogeneous even though the thickness of the cementite lamella is very thin (~10 nm). More specifically, chromium and manganese are slightly enriched around the interface (that is, depleted at the center part), while carbon is depleted in the vicinity of the interface, as shown in Figure 10(g,h). It should be noted that the local enrichment/depletion of elements is only observed inside the cementite lamella and not in the surrounding ferrite matrix. Similar heterogeneous element distributions in cementite lamella have been confirmed by a 3D atom probe analysis [41]. The analysis of element distribution around the side surface of a cementite lamella (corresponding to the growth front at transformation) is shown in Figure 11 ((a) STEM image, (b–f) element distribution maps ((b) carbon, (c) silicon, (d) chromium, (e) manganese, and (f) iron), and (g,h) concentration profiles across the side surface. We can find that the concentration profiles at the side surface are not sharp compared to those at the lamellar interface between ferrite and cementite (Figure 10). The region with gradual increase (carbon, manganese, and chromium)/decrease (silicon) of concentration toward the cementite ranges about 366 nm (from ‘B’ to ‘A’ in Figure 11). The STEM image of Figure 11(a) indicates that the side surface plane of cementite lamella would not be parallel to the incident electron beam direction. The side surface region inclined to the foil plane extends from ‘C’ to ‘D’, as estimated by the contrast change of cementite (Figure 11(a)). Its length is obviously shorter than the region with a gradual increase/decrease in concentration (from ‘B’ to ‘A’), suggesting that the broad concentration change at the side surface of cementite lamella is an intrinsic phenomenon.

## 4. Discussion

As described in the Introduction section, pearlite, when nucleated on a clean austenite grain boundary with an eutectoid composition, follows the Pitsch–Petch relationship. Conversely, pearlite that nucleates from hyper-eutectoid cementite and pro-eutectoid ferrite exhibits the Bagaryatskii and Isaichev relationships, respectively. As shown in Figures 1, 5, and 8, the observed orientation relationship in the present study is the Pitsch–Petch relationship. This could be because the microstructure exhibited almost fully pearlite due to air-cooling treatment, even though the present study used medium-carbon steel (0.62 % C), which is off-eutectoid in composition. However, this result is limited to the analyzed areas and does not imply that all air-cooled pearlites satisfy the Pitsch–Petch relationship.

Adachi et al. [18,21] performed 3D characterization of cementite lamellae in a eutectoid steel and reported that non-continuous regions are always presented in the naturally grown cementite lamella. Our 3D observations also demonstrated that a cementite lamella is not continuous and contains several non-continuous regions where ferrite protrudes into cementite lamella (Figures 2 and 4). The formation mechanism of these non-continuous regions present in the cementite lamella still remains unclear. The analysis on element distribution around the side surface of cementite lamella (corresponding to the growth front at transformation) shown in Figure 11 indicates that there is no significant chemical inhomogeneity, except for a broad concentration change, at the side surface. Therefore, we can conclude that local chemical inhomogeneity is not the cause of the discontinuous morphology of cementite lamella. Because the long axis direction of the non-continuous region is nearly identical in each cementite lamella as well as in the neighboring cementite lamellae (Figures 2 and 4), the shape of the non-continuous region would retain its original shape to some extent at transformation, rather than being formed during the subsequent cooling. Previous studies have observed growth ledges in pearlite structures [42,43]. Hackney and Shiflet proposed that the ledge mechanism may contribute to the synchronous growth of cementite and ferrite structures [42]. These results strongly suggest the presence of a crystallographically preferred growth direction in pearlite structures. We can consider that the long axis direction of the non-continuous region could be parallel to the preferential growth direction of cementite lamella. Several previous studies reported that the growth direction of precipitates tended to be parallel to an invariant line (or near invariant line) between matrix and precipitate [44–49]. Zhou and Shiflet [43] reported that, regardless of the macroscopic interfacial plane of the cementite lamella, its atomistic habit plane corresponds to ${\left(0\;1\;0\right)}_{\theta }//\;(\overset{ˉ}{2}\;\overset{ˉ}{1}\;5{)}_{\alpha }$, as long as the Pitsch–Petch relationship is satisfied. This implies that the interface of the cementite lamella exhibits a step structure, with terrace planes of ${\left(0\;1\;0\right)}_{\theta }//\;(\overset{ˉ}{2}\;\overset{ˉ}{1}\;5{)}_{\alpha }$, and that the macroscopic interface plane varies depending on the step spacing. Therefore, we can consider that the invariant lines on the atomistic habit plane of ${\left(0\;1\;0\right)}_{\theta }//\;(\overset{ˉ}{2}\;\overset{ˉ}{1}\;5{)}_{\alpha }$ plays an important role, even when the macroscopic interface plane is not exactly parallel to the atomistic habit plane. In order to determine the invariant line at the interface between ferrite and cementite, two simplifying assumptions were made in the present study; (i) the atomistic habit plane is ${\left(0\;1\;0\right)}_{\theta }//\;(\overset{ˉ}{2}\;\overset{ˉ}{1}\;5{)}_{\alpha }$ which corresponds to the parallel planes in the Pitsch–Petch relationship, as reported previously [28,50], and (ii) although ${\left[1\;3\;1\right]}_{\alpha }$ and ${\left[3\;\overset{ˉ}{1}\;1\right]}_{\alpha }$ are not exactly perpendicular in reality, the orthogonal principal distortions in the atomistic habit plane can be expressed as:

(1) $$\eta_{1}=\frac{\left|a_{\theta }{\left[100\right]}_{\theta }\right|}{\left|\frac{a_{\alpha }}{2}{\left[131\right]}_{\alpha }\right|}$$

(2) $$\eta_{2}=\frac{\left|c_{\theta }{\left[0\;0\;1\right]}_{\theta }\right|}{\left|\frac{a_{\alpha }}{2}{\left[3\;\overset{ˉ}{1}\;1\right]}_{\alpha }\right|}$$

According to the paper by Kato [48,51], the normalized invariant line vector can be expressed using the principal distortions in an orthogonal coordinate system normalized by the lattice constants of cementite;

(3) $$L_{I}=\left[\pm {\left|\frac{\eta_{2}^{2}-1}{\eta_{2}^{2}-\eta_{1}^{2}}\right|}^{1/2}0\pm {\left|\frac{1-\eta_{1}^{2}}{\eta_{2}^{2}-\eta_{1}^{2}}\right|}^{1/2}\right]$$

By using the lattice parameters of cementite and ferrite obtained by neutron diffraction (*a*_*θ*_ = 0.507 nm, *b*_*θ*_ = 0.676 nm, *c*_*θ*_ = 0.451 nm, and *a*_*α*_ = 0.287 nm), we determined the invariant line vectors as *L*_*I1*_ = [0.695 0 0.718] and *L*_*I2*_ = [$\bar{0.695}$ 0 0.718]. The corresponding normalized lattice vectors of cementite are *L*_*I1*_ = [0.652 0 0.758]_θ_ and *L*_*I2*_ = [$\bar{0.652}$ 0 0.758]_θ_. These invariant line vectors have been plotted on the standard stereographic projection of Figure 4(c). The result indicates that the projection of invariant line 1 onto the macroscopic interfacial plane determined by two-surface trace analysis approximately aligns with growth direction 1 (ranging from [2 1 1]_θ_ to [2 1 2]_θ_), with a deviation angle of 13° ~ 24°. Conversely, growth direction 2 is approximately [0 0 1]_θ_ and $[3\;\overset{ˉ}{1}\;1{]}_{\alpha }$, exhibiting a misorientation within the range of 4° ~ 10°. These directions are close to the parallel direction in the Pitsch–Petch relationship. Consequently, we propose that the growth direction of cementite lamella tends to align with the invariant line between cementite and ferrite, as well as the parallel direction in the Pitsch–Petch relationship. Amemiya et al. [52] reported that the transition of orientation relationships in pearlite is consistent with a selection mechanism governed by the requirement to form invariant lines between ferrite and cementite. Therefore, their results support the idea proposed in the present study that the invariant line plays an important role for the pearlite transformation.

Even though a nodule has been traditionally defined as a region with identical orientation of ferrite [12], we have shown that the colony boundaries inside the nodule have a small misorientation of ferrite ranging from 1.81° to 8.97° in the observed area (Figure 5(c,d)). Therefore, the colony boundaries not only change the interface normal of cementite lamella but also the ferrite orientation slightly. One can assume that the colony boundaries are formed by the impingement of independently growing pearlite region. However, the cementite lamella has an orientation relationship with that of the adjacent colony, and some of the cementite lamellae are interconnected with each other across the colony boundary, as shown in Figure 5(c–e). This indicates that the cementite lamellae change their interface normal at the colony boundaries while maintaining coherency with the existing cementite to a certain degree. As reported by Nakada et al. [53], we can consider that the growth direction changes at colony boundaries to accommodate the accumulated misfit strain between ferrite and cementite.

Our observation results, depicted in Figures 6 and 7, clearly indicate that the orientation change in a colony is discontinuous with low-angle boundaries (2° ~ 8°), and consistent with the EBSD analysis results reported by Walentek et al. [16]. Some of the low-angle boundaries exhibit a peculiar morphology, that is a staircase-like shape (Figures 6(b) and 7). It is difficult to imagine that such staircase-like boundaries were introduced after the completion of pearlite transformation to accommodate the accumulated misfit strain. Rather, these boundaries would correspond to the prior interphase boundary between pearlite and austenite matrix, and they provide evidence for the existence of a preferential growth direction of pearlite. Previous studies [42,43] proposed that the pearlite growth occurs through the migration of steps laterally across the growth front. The accumulation of misfit strain as well as the solute drag effect induced by segregation of alloying elements at the interphase boundary between ferrite and austenite could act as a back stress for pearlite transformation [54,55], resulting in the temporary stopping of the growth. Once the growth of pearlite is retarded, accommodation of strain and element partitioning could occur around the interphase boundary between the pearlite and austenite matrix. Additional growth can occur due to the increase in driving force resulting from subsequent cooling as well as the accommodation of misfit strain. Then, the prior interphase boundary between pearlite and austenite matrix is retained as a low-angle boundary with slight depletion of chromium and enrichment of silicon inside the pearlite (Figure 9). Moreover, as shown in Figure 8, the orientation relationship between ferrite and cementite is slightly changed at the low-angle boundary inside a colony; the upper colony exhibits [0 0 1]_θ_ // $[3\;\overset{ˉ}{1}\;1{]}_{\alpha }$, while the lower colony satisfies rather [1 0 0]_θ_ // [1 3 1]_α_. Assuming that the growth direction of pearlite was from lower to upper, the growth of the lower pearlite colony keeping the relationship of [1 0 0]_θ_//[1 3 1]_α_ would accumulate the misfit strain. We speculate that once the accumulated misfit strain exceeds a certain value, the parallel direction relationship changes from [1 0 0]_θ_//[1 3 1]_α_ (lower colony) to [0 0 1]_θ_//$[3\;\overset{ˉ}{1}\;1{]}_{\alpha }$ (upper colony) to accommodate the accumulated strain while keeping the nearly identical orientation relationship. Concerning the deviation of the [0 1 0]_θ_ direction from the $[\overset{ˉ}{2}\;\overset{ˉ}{1}\;5{]}_{\alpha }$ direction (Figure 8(c)), we do not believe that this results from measurement errors, as the measurement points within each region (upper and lower colonies) are not widely scattered. Three possibilities can be considered: (1) an intrinsic orientation relationship accompanied by a non-negligible deviation of the [0 1 0]_θ_ direction from the $[\overset{ˉ}{2}\;\overset{ˉ}{1}\;5{]}_{\alpha }$ direction; (2) a slight orientation change during subsequent cooling after the transformation, possibly resulting from partial spheroidization of the cementite lamella; and (3) inevitable elastic deformation induced during the fabrication process of the TEM specimen.

As illustrated in Figure 10, the element distribution inside the cementite lamella is not homogeneous; manganese and chromium are enriched at the lamellar interface, while carbon is depleted at the lamellar interface. Chance and Ridley [56] reported that the partitioning coefficient for pearlite is considerably smaller compared to that in equilibrium. This means that the condition for local equilibrium at the transformation is not fully satisfied, particularly at low temperatures. We can consider that pearlite transformation occurs without complete partitioning of alloying elements (that is, no-partition mode or negligible-partitioning local equilibrium mode), and the alloying elements are partitioned during cooling after the formation of pearlite (the present study employed air-cooling for pearlite transformation). Hutchison et al. [57] studied the partitioning behavior of pearlite in Fe-Mn-C alloy and found that the manganese content inside cementite increased with longer isothermal-holding periods, particularly during holding in the austenite/ferrite/cementite three-phase temperature range. Moreover, Zhang et al. [55] reported no macroscopic partitioning of manganese across the interphase boundary between pearlite and austenite in Fe-Mn-C alloy. Even when pearlite forms with partitioning of alloying elements, variations in the equilibrium partitioning coefficient with temperature could lead to re-partitioning of alloying elements during cooling. This process results in an inhomogeneous element distribution inside the cementite. However, we should note here that the observed inhomogeneous element distribution along the thickness direction of the cementite lamella presented in Figure 10 cannot be explained solely by the incomplete partitioning behavior of alloying elements at transformation. An incomplete partitioning situation results in simultaneous enrichment (or depletion) in the cementite and depletion (or enrichment) in the ferrite matrix across the interface. For example, the enrichment of manganese around the interface on the cementite side should bring the depletion in the ferrite matrix side. As shown in Figure 10, although manganese and chromium are enriched and carbon is depleted around the interface, no depletion or enrichment of elements on the ferrite matrix side has been observed. Based on the previous reports [16,20] as well as the results presented in Figure 6, the misfit strain at the interface of cementite lamella appears significant enough to induce variations in crystal orientation. We surmise that the segregation of manganese and chromium at the interface, which decreases interfacial energy, also contributes to the inhomogeneous element distribution of cementite lamella.

To date, it has been simply considered that the strength of pearlite is primarily associated with an interlamellar spacing [8,58–62]. On the other hand, the effective structural unit for the toughness of pearlite remains a topic of controversy: interlamellar spacing [59,60], nodule size [8,9], etc. We suggest that the intrinsic defects associated with the formation of cementite (Figures 2 and 4) and localized plastic deformation (Figures 5–7) could potentially serve as initiation sites for fracture. The fundamentals behind the inherently low impact-toughness, a critical issue in the as-transformed pearlite [8–11], could lie in these intrinsic defects. Moreover, the local concentration of alloying elements around the interface of cementite lamella (Figure 10) could influence the plastic deformation behavior and brittle fracture behavior (particularly, decohesion of lamellar interface). Accordingly, we can propose that in order to precisely understand the origin of macroscopic mechanical properties of pearlite, it is crucial to elucidate the relationship between its substructures and mechanical properties.

## 5. Conclusions

We investigated the morphology, substructure, crystallography, and local element distribution of as-transformed (air-cooled) pearlite in medium-carbon steel using FIB-SEM serial sectioning and TEM/STEM analysis. The following conclusions were drawn.

1. The 3D analysis revealed that the cementite lamella did not exhibit a completely continuous morphology, and the long axis direction of the non-continuous region was nearly identical within each cementite lamella and among the adjacent cementite lamellae. Cementite lamella does not have a unique orientation for its macroscopic interfacial plane, and the orientation of the measured interfacial plane varies within a range from (0 1 0)_θ_ to $\left(\bar{1}\;2\;0\right)$_θ_ and $(\overset{ˉ}{2}\;\overset{ˉ}{1}\;5{)}_{\alpha }$ to $(\overset{ˉ}{1}\;\overset{ˉ}{1}\;1{)}_{\alpha }$. Assuming that the long axis direction of the non-continuous region could be parallel to the preferential growth direction of cementite lamella, we identified that one growth direction ranged from [2 1 1]_θ_ to [2 1 2]_θ_ and around [1 1 2]_α_, while the other was primarily around [0 0 1]_θ_ and $[3\;\overset{ˉ}{1}\;1{]}_{\alpha }$ These growth directions of cementite lamella tended to align with the invariant line between cementite and ferrite, as well as the parallel direction in the Pitsch–Petch relationship.
2. The STEM analysis revealed that the colony boundaries inside the nodule exhibited a small misorientation of ferrite, and the cementite lamellae had an orientation relationship with those of the adjacent colony. Furthermore, some of the cementite lamellae were interconnected with each other across the colony boundary. These results suggest that the cementite lamellae alter its interface normal at colony boundaries while still maintaining coherency with the existing cementite.
3. Even within a single colony, the orientation changed discontinuously, forming low-angle boundaries (2° ~ 8°). This indicates that local plastic deformation occurred during the pearlite transformation. Some of the low-angle boundaries exhibited a staircase-like shape, which would correspond to the prior interphase boundary between pearlite and austenite matrix. We found that the orientation relationship between ferrite and cementite changed slightly at the low-angle boundary within a colony. The upper colony exhibited [0 0 1]_θ_ // $[3\;\overset{ˉ}{1}\;1{]}_{\alpha }$, while the lower colony satisfied rather [1 0 0]_θ_ // [1 3 1]_α_. We speculated that when the accumulated misfit strain exceeds a certain value, the parallel direction relationship changes from [1 0 0]_θ_ // [1 3 1]_α_ to $[0\;0\;1]//[3\;\overset{ˉ}{1}\;1{]}_{\alpha }$ (or vice versa) to accommodate the accumulated strain while maintaining the nearly identical orientation relationship.
4. The concentration inside cementite lamella was not completely homogeneous; specifically, manganese and chromium were enriched at the interface, while carbon was depleted at the interface. We surmised that the inhomogeneous element distribution in cementite lamella could be attributed to the incomplete partitioning behavior of alloying elements at transformation, as well as their segregation at the interface of cementite lamella, aiming to decrease interfacial energy.

## Supplementary Material

Supplemental Material

## Data Availability

The raw/processed data required to reproduce these findings cannot be shared at this time, as the data also form part of an ongoing study.

## AppendicesAppendix A

The pillar for FIB-SEM serial sectioning was lifted out from the bulk sample and, from the beginning, mounted onto a copper mesh that fits the TEM specimen holder before performing the FIB-SEM serial sectioning (Figure A1(a–d)). As shown in Figure A1(e,f), the last section for FIB-SEM serial sectioning and the film plane of TEM/STEM specimen are almost identical (the difference is approximately 250 nm). We understand that achieving perfect alignment of specimen geometry between the FIB-SEM serial sectioning and TEM/STEM observation is challenging. However, the SEM image of the final section of FIB-SEM serial sectioning and STEM image (Figure A1(g,h)) demonstrate that the alignment difference between these analyses is negligibly small.

## Appendix B

Using the orientation matrixes of neighboring ferrite (***F***_***i***_) and cementite (***C***_***i***_), we calculated the independent rotation matrix (***R***_***i***_) for each orientation pairs (22 pairs in total, that is *i* = 1 ~ 22) from the following equation:

(B1) $$R_{i}=F_{i}\;{C_{i}}^{-1}$$

Then, the ferrite orientations exactly parallel to [0 1 0]_θ_, [0 0 1]_θ_, and [1 0 0]_θ_ of cementite were calculated with the rotation matrix, as described in Eqs. B2, B3, and B4.

(B2) $$\vec{F_{i}}(//[0\;1\;0{]}_{\theta })=R_{i}{\left[\begin{array}{c} 0\\ 1\\ 0 \end{array}\right]}_{\theta }$$

(B3) $$\vec{F_{i}}(//[0\;0\;1{]}_{\theta })=R_{i}{\left[\begin{array}{c} 0\\ 0\\ 1 \end{array}\right]}_{\theta }$$

(B4) $$\vec{F_{i}}(//[1\;0\;0{]}_{\theta })=R_{i}{\left[\begin{array}{c} 1\\ 0\\ 0 \end{array}\right]}_{\theta }$$

Then, the $\vec{F_{i}}$(//[0 1 0]_θ_), $\vec{F_{i}}$(//[0 0 1]_θ_), and $\vec{F_{i}}$(//[1 0 0]_θ_) for 22 pairs of ferrite and cementite are plotted in Figure 8(c–e). That is, each plot in Figure 8(c–e) represents the orientation relationship, with deviations of [0 1 0]_θ_ from $[\overset{ˉ}{2}\;\overset{ˉ}{1}\;5{]}_{\alpha }$ (Figure 8(c)), ${\left[0\;0\;1\right]}_{\theta }$ from $[3\;\overset{ˉ}{1}\;1{]}_{\alpha }$, (Figure 8(d)), and ${\left[1\;0\;0\right]}_{\theta }$ from $[\;1\;\;3\;\;1{]}_{\alpha }$ (Figure 8(e)), for the corresponding ferrite and cementite pair shown in Figure 8(a).

## References

[1] Li Y, Raabe D, Herbig M, et al. Segregation stabilizes nanocrystalline bulk steel with near theoretical strength. Phys Rev Lett. 2014;113(10):106104. doi: 10.1103/PhysRevLett.113.106104 25238372

[2] Yamasaki S. The microstructure and mechanical properties of drawn and aged pearlitic steel wires. Mater Sci Technol. 2018;34(1):1–19. doi: 10.1080/02670836.2017.1407542

[3] Zhang XD, Hansen N, Godfrey A, et al. Structure and strength of sub-100 nm lamellar structures in cold-drawn pearlitic steel wire. Mater Sci Technol. 2018;34(7):794–808. doi: 10.1080/02670836.2018.1440155

[4] Lee KM, Polycarpou AA. Wear of conventional pearlitic and improved bainitic rail steels. Wear. 2005;259(1–6):391–399. doi: 10.1016/j.wear.2005.02.058

[5] Korda A, Miyashita Y, Mutoh Y, et al. Fatigue crack growth behavior in ferritic–pearlitic steels with networked and distributed pearlite structures. Int J Fatigue. 2007;29(6):1140–1148. doi: 10.1016/j.ijfatigue.2006.09.008

[6] Kim JS, Lee YH, Lee DL, et al. Microstructural influences on hydrogen delayed fracture of high strength steels. Mater Sci Eng A. 2009;505(1–2):105–110. doi: 10.1016/j.msea.2008.11.040

[7] Ronevich JA, Somerday BP, San Marchi CW. Effects of microstructure banding on hydrogen assisted fatigue crack growth in X65 pipeline steels. Int J Fatigue. 2016;82:497–504. doi: 10.1016/j.ijfatigue.2015.09.004

[8] Hyzak JM, Bernstein IM. The role of microstructure on the strength and toughness of fully pearlitic steels. Metall Trans A. 1976;7(8):1217–1224. doi: 10.1007/BF02656606

[9] Park YJ, Bernstein IM. The process of crack initiation and effective grain size for cleavage fracture in pearlitic eutectoid steel. Metall Trans A. 1979;10(11):1653–1664. doi: 10.1007/BF02811698

[10] Khiratkar VN, Mishra K, Srinivasulu P, et al. Effect of inter-lamellar spacing and test temperature on the charpy impact energy of extremely fine pearlite. Mater Sci Eng A. 2019;754:622–627. doi: 10.1016/j.msea.2019.03.121

[11] Behera S, Barik RK, Sk MB, et al. Recipe for improving the impact toughness of high-strength pearlitic steel by controlling the cleavage cracking mechanisms. Mater Sci Eng A. 2019;764:138256. doi: 10.1016/j.msea.2019.138256

[12] Bhadeshia HKDH. Theory of transformations in steels. New York (NY): CRC Press; 2021.

[13] Tian YL, Kraft RW. Kinetics of pearlite spheroidization. Metall Trans A. 1987;18(8):1359–1369. doi: 10.1007/BF02646650

[14] Tian YL, Kraft RW. Mechanisms of pearlite spheroidization. Metall Trans A. 1987;18(8):1403–1414. doi: 10.1007/BF02646654

[15] Werner E. The growth of holes in plates of cementite. Mater Sci Eng A. 1991;132:213–223. doi: 10.1016/0921-5093(91)90377-Y

[16] Walentek A, Seefeldt M, Verlinden B, et al. Electron backscatter diffraction on pearlite structures in steel. J Microscopy. 2006;224(3):256–263. doi: 10.1111/j.1365-2818.2006.01702.x 17210058

[17] Takahashi T, Ponge D, Raabe D. Investigation of orientation gradients in pearlite in hypoeutectoid steel by use of orientation imaging microscopy. Steel Res Int. 2007;78(1):38–44. doi: 10.1002/srin.200705857

[18] Adachi Y, Morooka S, Nakajima K, et al. Computer-aided three-dimensional visualization of twisted cementite lamellae in eutectoid steel. Acta Mater. 2008;56(20):5995–6002. doi: 10.1016/j.actamat.2008.08.017

[19] Zaefferer S, Wright SI, Raabe D. Three-dimensional orientation microscopy in a focused ion beam–scanning electron microscope: a new dimension of microstructure characterization. Metall Mater Trans A. 2008;39(2):374–389. doi: 10.1007/s11661-007-9418-9

[20] Nakada N, Koga N, Tsuchiyama T, et al. Crystallographic orientation rotation and internal stress in pearlite colony. Scr Mater. 2009;61(2):133–136. doi: 10.1016/j.scriptamat.2009.03.028

[21] Wang YT, Adachi Y, Nakajima K, et al. Quantitative three-dimensional characterization of pearlite spheroidization. Acta Mater. 2010;58(14):4849–4858. doi: 10.1016/j.actamat.2010.05.023

[22] Endo S, Miyazawa N, Nakada N, et al. Formation mechanism of pearlite colony by multiple orientation relationships between ferrite and cementite. ISIJ Int. 2022;62:291–298. doi: 10.2355/isijinternational.ISIJINT-2021-332

[23] Petch NJ. The orientation relationships between cementite and α-iron. Acta Cryst. 1953;6(1):96. doi: 10.1107/S0365110X53000260

[24] Pitsch W. Der orientierungszusammenhang zwischen zementit und ferrit im perlit. Acta Metall. 1962;10(1):79–80. doi: 10.1016/0001-6160(62)90190-6

[25] Bagaryatsky YA. Possible mechanism of martensite decomposition. Dokl Akad Nauk SSSR. 1950;73:1161–1164.

[26] Isaichev IV. Orientation of cementite in tempered carbon steel. Zh Tekh Fiz. 1947;17:835–838.

[27] Dippenaar RJ, Honeycombe RW. Crystallography and nucleation of pearlite. Proc R Soc Lond A. 1973;333:455–467.

[28] Zhou DS, Shiflet GJ. Ferrite–cementite crystallography in pearlite. Metall Trans A. 1992;23(4):1259–1269. doi: 10.1007/BF02665057

[29] Nakajima K, Apel M, Steinbach I. The role of carbon diffusion in ferrite on the kinetics of cooperative growth of pearlite: a multi-phase field study. Acta Mater. 2006;54(14):3665–3672. doi: 10.1016/j.actamat.2006.03.050

[30] Steinbach I, Apel M. The influence of lattice strain on pearlite formation in Fe–C. Acta Mater. 2007;55(14):4817–4822. doi: 10.1016/j.actamat.2007.05.013

[31] Mouri M, Tsukada Y, Koyama T. Phase-field simulation of the effect of interphase boundary diffusion on pearlite transformation in Fe–C system. Tetsu-To-Hagane. 2019;105:305–313.

[32] Mushongera LT, Amos PGK, Nestler B, et al. Phase-field simulations of pearlitic divergence in Fe–C–Mn steels. Acta Mater. 2018;150:78–87. doi: 10.1016/j.actamat.2018.02.059

[33] Yamanaka A. Phase-field modeling and simulation of solid-state phase transformations in steels. ISIJ Int. 2023;63:395–406. doi: 10.2355/isijinternational.ISIJINT-2022-343

[34] Shibata A, Gutierrez-Urrutia I, Nakamura A, et al. Multi-scale three-dimensional analysis on local arrestability of intergranular crack in high-strength martensitic steel. Acta Mater. 2022;234:118053. doi: 10.1016/j.actamat.2022.118053

[35] Shibata A, Miyamoto G, Morito S, et al. Substructure and crystallography of lath martensite in as-quenched interstitial-free steel and low-carbon steel. Acta Mater. 2023;246:118675. doi: 10.1016/j.actamat.2023.118675

[36] Shibata A, Gutierrez-Urrutia I, Nakamura A, et al. Three-dimensional propagation behavior of hydrogen-related intergranular cracks in high-strength martensitic steel. Int J Hydrog Energy. 2023;48(88):34565–34574. doi: 10.1016/j.ijhydene.2023.05.211

[37] Shibata A, Gutierrez-Urrutia I, Nakamura A, et al. Local crack arrestability and deformation microstructure evolution of hydrogen-related fracture in martensitic steel. Corros Sci. 2024;233:112092. doi: 10.1016/j.corsci.2024.112092

[38] Ueji R, Kimura Y, Inoue T. Preferable resistance against hydrogen embrittlement of pearlitic steel deformed by caliber rolling. ISIJ Int. 2022;62:368–376. doi: 10.2355/isijinternational.ISIJINT-2021-429

[39] Hara T, Tsuchiya K, Tsuzaki K, et al. Application of orthogonally arranged FIB–SEM for precise microstructure analysis of materials. J Alloys Compd. 2013;577:S717–S721. doi: 10.1016/j.jallcom.2012.02.019

[40] Hansen N. New discoveries in deformed metals. Metall Mater Trans A. 2001;32(12):2917–2935. doi: 10.1007/s11661-001-0167-x

[41] Li ZH, Sasaki TT, Ueji R, et al. Role of deformation on the hydrogen trapping in the pearlitic steel. Scr Mater. 2024;241:115859. doi: 10.1016/j.scriptamat.2023.115859

[42] Hackney SA, Shiflet GJ. The pearlite-austenite growth interface in an Fe-0.8C-12Mn alloy. Acta Metall. 1987;35(5):1007–1017. doi: 10.1016/0001-6160(87)90048-4

[43] Zhou DS, Shiflet GJ. Interfacial steps and growth-mechanism in ferrous pearlites. Metall Trans A. 1991;22(6):1349–1365. doi: 10.1007/BF02660668

[44] Dahmen U. Orientation relationships in precipitation systems. Acta Metall. 1982;30(1):63–73. doi: 10.1016/0001-6160(82)90045-1

[45] Dahmen U, Ferguson P, Westmacott KH. Invariant line strain and needle-precipitate growth directions in Fe–Cu. Acta Metall. 1984;32(5):803–810. doi: 10.1016/0001-6160(84)90153-6

[46] Luo CP, Weatherly GC. The invariant line and precipitation in a Ni–45 wt-percent Cr alloy. Acta Metall. 1987;35(8):1963–1972. doi: 10.1016/0001-6160(87)90025-3

[47] Kato M, Wada M, Sato A, et al. Epitaxy of cubic-crystals on (001) cubic substrates. Acta Metall. 1989;37(3):749–756. doi: 10.1016/0001-6160(89)90001-1

[48] Kato M. Invariant-plane and invariant-line deformation criteria and their application to interface crystallography. Mater Trans JIM. 1992;33(2):89–96. doi: 10.2320/matertrans1989.33.89

[49] Luo CP, Dahmen U, Westmacott KH. Morphology and crystallography of Cr precipitates in a Cu–0.33 wt-percent Cr alloy. Acta Metall Mater. 1994;42(6):1923–1932. doi: 10.1016/0956-7151(94)90017-5

[50] Kosaka M, Ushioda K, Teshima T, et al. Three-dimensional quantitative evaluation of the lamellar curvature in pearlitic steel based on an orientation analysis of cementite. ISIJ Int. 2022;62:299–306. doi: 10.2355/isijinternational.ISIJINT-2021-194

[51] Kato M. Simple criteria for epitaxial relationships between FCC and BCC crystals. Mater Sci Eng A. 1991;146(1–2):205–216. doi: 10.1016/0921-5093(91)90278-U

[52] Amemiya Y, Nakada N, Morooka S, et al. Dynamic accommodation of internal stress and selection of crystallographic orientation relationship in pearlite. ISIJ Int. 2022;62:282–290. doi: 10.2355/isijinternational.ISIJINT-2021-164

[53] Nakada N, Kato M. Internal stress and elastic strain energy in pearlite and their accommodation by misfit dislocations. ISIJ Int. 2016;56(10):1866–1873. doi: 10.2355/isijinternational.ISIJINT-2016-256

[54] Hillert M. On theories of growth during discontinuous precipitation. Metall Trans. 1972;3(11):2729–2741. doi: 10.1007/BF02652840

[55] Zhang YJ, Umeda T, Morooka S, et al. Pearlite growth kinetics in Fe-C-Mn eutectoid steels: quantitative evaluation of energy dissipation at pearlite growth front via experimental approaches. Metall Mater Trans A. 2024;55(10):3921–3936. doi: 10.1007/s11661-024-07518-1

[56] Chance J, Ridley N. Chromium partitioning during isothermal transformation of a eutectoid steel. Metall Trans A. 1981;12(7):1205–1213. doi: 10.1007/BF02642334

[57] Hutchinson CR, Hackenberg RE, Shiflet GJ. The growth of partitioned pearlite in Fe–C–Mn steels. Acta Mater. 2004;52(12):3565–3585. doi: 10.1016/j.actamat.2004.04.010

[58] Gladman T, Id M, Pickering FB. Some aspects of the structure–property relationships in high-carbon ferrite–pearlite steels. J Iron Steel Inst. 1972;210:916–930.

[59] Marder AR, Bramfitt BL. The effect of morphology on the strength of pearlite. Metall Trans A. 1976;7(3):365–372. doi: 10.1007/BF02642832

[60] Lewandowski JJ, Thompson AW. Microstructural effects on the cleavage fracture stress of fully pearlitic eutectoid steel. Metall Mater Trans A. 1986;17(10):1769–1786. doi: 10.1007/BF02817275

[61] Taleff EM, Syn CK, Lesuer DR, et al. Pearlite in ultrahigh carbon steels: heat treatments and mechanical properties. Metall Mater Trans A. 1996;27(1):111–118. doi: 10.1007/BF02647751

[62] Taleff EM, Lewandowski JJ, Pourladian B. Microstructure–property relationships in pearlitic eutectoid and hypereutectoid carbon steels. JOM. 2002;54(7):25–30. doi: 10.1007/BF02700982

